# Autophagic Mechanism in Anti-Cancer Immunity: Its Pros and Cons for Cancer Therapy

**DOI:** 10.3390/ijms18061297

**Published:** 2017-06-19

**Authors:** Ying-Ying Li, Lynn G. Feun, Angkana Thongkum, Chiao-Hui Tu, Shu-Mei Chen, Medhi Wangpaichitr, Chunjing Wu, Macus T. Kuo, Niramol Savaraj

**Affiliations:** 1 Department of Medicine, University of Miami Miller School of Medicine, Miami, FL 33136, USA; yli4@med.miami.edu; 2 Sylvester Comprehensive Cancer Center, University of Miami Miller School of Medicine, Miami, FL 33136, USA; lfeun@med.miami.edu; 3 Laboratory of Environmental Toxicology, Chulabhorn Research Institute, Thailand Chulabhorn Graduate Institute, Laksi, Bangkok 10210, Thailand; nucleoside.analogue@gmail.com; 4 Department of Neurosurgery, Wan Fang Hospital, Taipei Medical University, Taipei 116, Taiwan; s97324529@mail1.ncnu.edu.tw (C.-H.T.); nschenis@gmail.com (S.-M.C.); 5 Department of Surgery, University of Miami Miller School of Medicine, Miami, FL 33136, USA; mwangpaichitr@med.miami.edu; 6 Division of Hematology and Oncology, Miami Veterans Affairs Healthcare System, Miami, FL 33125, USA; chunjing.wu@va.gov; 7 Department of Molecular Pathology, University of Texas M. D. Anderson Cancer Center, Houston, TX 77030, USA; tkuo@mdanderson.org

**Keywords:** autophagy, anticancer immunity, immunogenicity, autophagy antagonist, tumor microenvironment

## Abstract

Autophagy, a self-eating machinery, has been reported as an adaptive response to maintain metabolic homeostasis when cancer cells encounter stress. It has been appreciated that autophagy acts as a double-edge sword to decide the fate of cancer cells upon stress factors, molecular subtypes, and microenvironmental conditions. Currently, the majority of evidence support that autophagy in cancer cells is a vital mechanism bringing on resistance to current and prospective treatments, yet whether autophagy affects the anticancer immune response remains unclear and controversial. Accumulated studies have demonstrated that triggering autophagy is able to facilitate anticancer immunity due to an increase in immunogenicity, whereas other studies suggested that autophagy is likely to disarm anticancer immunity mediated by cytotoxic T cells and nature killer (NK) cells. Hence, this contradiction needs to be elucidated. In this review, we discuss the role of autophagy in cancer cells per se and in cancer microenvironment as well as its dual regulatory roles in immune surveillance through modulating presentation of tumor antigens, development of immune cells, and expression of immune checkpoints. We further focus on emerging roles of autophagy induced by current treatments and its impact on anticancer immune response, and illustrate the pros and cons of utilizing autophagy in cancer immunotherapy based on preclinical references.

## 1. Overview of Autophagy

**Figure 1:**
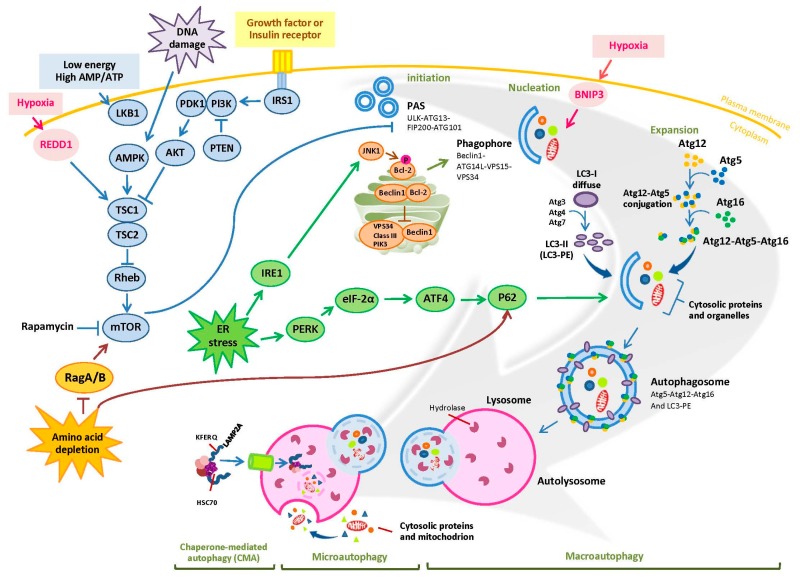
Diagram illustrates three types of autophagy, autophagosome formation, and autophagic signaling. Three types of autophagy, macroautophagy, microautophagy, and chaperon-mediated autophagy (CMA) have been found in eukaryotic cells. Macroautophagy is the most common type found in mammalian cells which proceeds through several steps including initiation, nucleation, expansion/elongation to form autophagosome, and fusion of autophagosome-lysosome to become autolysosome. These steps are performed by autophagy-related (Atg) proteins and other autophagic proteins, and regulated by different types of stress and signals, such as DNA damage, endoplasmic reticulum (ER) stress, growth factors or insulin, hypoxia, and amino acid depletion.

Autophagy, an essential mechanism responsible for sustaining metabolism in eukaryotic cells, has been known over 50 years. As reported by numerous studies, autophagy recycles intracellular components including proteins, ribosomes, and organelles (e.g., mitochondria, peroxisomes, endoplasmic reticulum, etc.), exerting different functions to maintain metabolic homeostasis, control proliferation, and ensure survival when cells confront environmental stress [1,2,3]. Currently, three major autophagic mechanisms have been discovered: macroautophagy, microautophagy, and chaperone-mediated autophagy (CMA) (Figure 1). Macroautophagy, representing the majority of autophagy, is a common process of degrading intracellular components for nutrient recovery through forming the phagophores (isolation membrane), sequestering them into double membrane-bound vacuoles (so-called autophagosomes), and fusing with lysosomes (also called autolysosomes). The detailed mechanisms of macroautophagy are summarized in Figure 1. Microautophagy is a process of nonselective degradation which directly engulfs cytoplasmic components and organelles into tubules or the lysosomes [4]. CMA, different from microautophagy, only contributes to degradation of soluble proteins possessing a KFERQ sequence motif. Subsequent to recognition by cytosolic heat shock protein of 70 kD (Hsc70), these proteins form a complex with Hsc70 and its chaperones for delivery to lysosomes where it interacts with lysosome-associated membrane protein (LAMP)-2A receptor, and hence undergo degradation [5].

In regard to molecular-based mechanism of macroautophagy, autophagy-related (Atg) proteins are the vital molecules implicated in autophagosome formation once receiving incoming autophagic signals (Figure 1). The first (initiation) step of autophagosome formation is governed by the complex of ULK-1–Atg13–FIP200. This complex is negatively regulated by mammalian target of rapamycin (mTOR) through phosphorylation of ULK. The second (phagophore nucleation) step is accomplished by class-III phosphatidylation 3-kinase (PI3K)-Beclin1 complex consisting of Beclin1, Atg14L, Vps 15, and Vps 34. The third (elongation or expansion) step is the Atg5–Atg12 formation along with Atg16 to become the Atg5–Atg12–Atg16 complex. Simultaneously, LC3 (Atg8)-I is cleaved by Atg4 and in turn conjugated with phosphatidylethanolamine (PE) by Atg3 and Atg7 to become LC3-II. The double-membrane-associated LC3-II is involved in phagophore enclosure resulting in autophagosome formation. Although the mechanism of autophagosome formation is well known, as described above, the key issue is why cells decide to undergo autophagy. The most well-known factors are those related to various stress signals.

It is known that under energy starvation, low cellular ATP/AMP ratio triggers the activation of LKB1–AMPK axis (an energy sensor) through phosphorylation. Activated AMPK directly initiates ULK1–Atg13–FIP200 complex-mediated autophagosome formation or through TSC1/2 dependent or independent mTOR inhibition [6]. Similar to energy starvation, DNA damage and hypoxia also contribute to activation of AMPK (Figure 1). In addition to AMPK activation, hypoxia is capable of suppressing mTOR via BCL2 Interacting Protein 3 (BNIP3) and regulated in development and DNA damage responses 1 (REDD1), resulting in macroautophagy [7,8]. Other signals regulating Atg-mediated autophagosome formation include growth factors such as insulin and their receptors, ER stress, and amino acid depletion. It is appreciated that when growth factors or insulin interact with their tyrosine kinase receptors, the binding triggers activation of class I PI3K, which produces PIP3 to recruit AKT. This process can be suppressed by phosphatase and tensin homologue (PTEN). Once AKT has been recruited, it is phosphorylated/activated by phosphoinositide-dependent kinase-1 (PDK1). Activated AKT then phosphorylates the TSC2 to abrogate formation of TSC1/2 complex. It is known that TSC2 complex inhibits mTOR activation to promote autophagy through its GTPase protein (GAP) activity toward small GTPase Rheb [9,10]. Hence, deactivated TSC1/2 increases the ability of mTOR to inhibit autophagy. On the other hand, ER stress and amino acid depletion have been reported to increase the level of p62, which serves as adaptor of autophagy and interacts with LC3 for autophagosome formation [11]. ER stress gives rise to elevated p62 (also known as sequestosome-1 (SQSTM1)) expression through activation of PERK-mediated eIF2α–ATF4 pathway [11], and also turns on ER transmembrane sensor inositol-requiring enzyme 1(IRE1) to activate Jun N-terminal kinase (JNK) [12]. JNK activation suppresses/phosphorylates anti-apoptotic protein BCL2, and then leads to dissociation of BCL2 from Beclin-1, resulting in autophagic influx [13]. Under amino acid starvation, Rag GTPase (RagA/B^GDP^-RagC/D^GTP^) heterodimers are unable to activate mTOR and consequently induce autophagy [14]. Recent studies further demonstrated that deprivation of leucine or arginine disrupts Rags-mediated mTOR activation through their sensors Sestrin2 and arginine sensor for mTORC1 (CASTOR1), and also increases AMPK activation [15,16,17,18].

Autophagy has been reported to play a crucial role in a range of disease including chronic liver disease, infectious disease, cardiovascular disease, neurodegenerative disease, obesity, and cancer [19,20]. In cancer cells, it has been considered as a double-edge sword to manipulate tumorigenesis, cell proliferation, and cell survival. Although many studies have uncovered the mechanisms leading to autophagic cascade, the factors which can regulate autophagy-mediated cell survival or cell death in cancer cell, tumor microenvironment, and immune surveillance remain unclear and controversial. Therefore, understanding the relationship between autophagy and these factors is needed for improvement of cancer immunotherapies or in combination with other treatments.

## 2. The Role of Autophagy in Cancer Cells: Survival or Death Signals?

Whether the role of autophagy is *pro* or *con* for cancer cells has been debating for many years. Some references elucidated that deficiency of autophagy results in tumorigenesis. For instance, in PTEN (+/−) deletion-driven tumor mouse models, down-regulation of LKB–AMPK expression resulted in a drastic acceleration of tumorigenesis through activation of mTOR [21]. Moreover, oncogenic BRAF has been reported to activate MAPK and its downstream ribosomal S6 kinase (RSK), which deactivates LKB–AMPK axis through phosphorylation of LKB at Ser428 and Ser325 and thereby hinders autophagy [22]; therefore, it has been considered as a tumor suppressor. Additionally, heterozygous disruption of *Beclin1* gene (also known as Atg6) in mice caused a high incidence of spontaneous tumors, such as hepatoma, B cell lymphoma, and lung adenocarcinoma. Clinical data have revealed that 40–75% of ovarian and prostate cancers that possess heterozygous disruption in *Beclin1* gene were related to aggressive phenotypes [23]. Collectively, autophagy-associated molecules are usually related to deterring tumor initiation, and hence deficiency of autophagy promotes tumorigenesis. However, heterozygous loss of *Beclin1* in mouse mammary gland delays breast cancer development [24]. Thus, the role of autophagy in tumor initiation is possibly cell context specific. Tumor cells have been known to utilize autophagic process upon confrontation with stress in order to avoid apoptosis, yet autophagy-dependent cell death appears in specific types of cancer cells when treated with certain anticancer therapeutic agents. These examples are discussed below.

### 2.1. Autophagy and Cancer Cell Survival

Cumulative evidence has demonstrated that autophagy mostly leads to cancer survival and resistance to therapeutic agents (Table 1). It remains unclear how autophagic process can either assist cell survival or result in cell death. However, it is doubtless that one type of stress requires autophagy to survive is nutrient deprivation stress. This includes glucose or amino acids starvation such as arginine, leucine, and others. Currently, it has been known that low glucose levels directly give rise to activation of AMPK, and glycolysis inhibition using 2-deoxyglucose (DG) results in ER stress. Both pathways confer autophagy-dependent survival to cells as evidenced by active LC3-I/II conversion [20,21]. The other nutrient, arginine, is regarded as an essential amino acid for cancer cells that do not express or express very low levels of argininosuccinate synthase 1 (ASS1), a key enzyme to synthesize arginine from citrulline. According to our and other studies, ASS1-deficient melanoma cells turn on AMPK-mediated autophagy to survive under arginine deprivation [18,25]. In reference to chemotherapeutic agents known to cause DNA damage (temozolomide and cisplatin), inhibition of DNA synthesis (5-fluorouracil (FU) and gemcitabine), and HDAC inhibition (SAHA), they induce growth inhibition and autophagy in order to survive [26,27,28,29,30,31,32]. Other agents which target signal transduction pathways due to specific gene mutation, amplification, and activation, such as erlotinib and gefitinib (EGFR mutation), imatinib (tyrosine kinase activation), vemurafenib and dabrafenib (BRAF mutaion), and trastuzumab (HER2 amplification) also give rise to autophagy-mediated cell survival [30,31,32,33,34,35,36,37,38]. Based on these evidence, the inhibitors against autolysosome formation such as chloroquine (CQ), hydroxy-chloroquine (HCQ), bafilomycin A, and 3-methyladenine (MA) have been examined in combination of these antitumor agents and have shown significant improvement secondary to induction of apoptosis in vitro. Furthermore, genetic interruption of autophagic proteins has been shown to elevate oxidative stress and increase sensitivity to inflammation-enhanced genetic instability [33]. Taken together, combination of these therapeutic agents with autophagy inhibitors may cause beyond abrogation of autophagy-dependent cell survival. Despite multiple studies uncovering that autophagy is a protective mechanism in response to these anticancer therapies and may contribute to acquired resistance, cancer cells may abandon autophagy in order to proliferate and metastasize once resistance is fully developed. For example, BRAF inhibitor-resistant melanoma cells which possess hyperactivation of ERK and AKT to overcome BRAF inhibition, yet they gradually lose autophagic proteins including Atg5 and AMPK while continuously exposed to BRAF inhibitor vemurafenib [34].

Since mTOR is the master regulator for cell growth/proliferation and plays a crucial role in autophagy, treatment with mTOR inhibitors, such as NVP-BEZ235, RAD001, and AZD8055, inhibits cell growth and reduces cell survival. The apoptotic effect is possibly through some different mechanisms (Table 1) and off-target effects [35]. Importantly, mTOR activity which is regulated by its multiple partners including raptor and rictor, is highly complex [36]. Altogether, the response to the various mTOR inhibitors mentioned above not only depends on dosage, but also on the levels of anti-apoptotic protein as well as the signaling pathways used for the cancer cells to thrive [35]. In certain cells (e.g., mouse embryonic fibroblasts (MEFs) with G12D mutation at KRAS) relying on extracellular proteins as their amino acid source, inhibition of mTORC1 under nutrient limited condition leads to an increase in endocytosed proteins catabolism and promotes tumor cell proliferation [37]. Thus, cell type and environment/stress the cells encounter also contribute to mTOR-meditated cell survival/death. Additionally, autophagy also has been reported to emerge in irradiated cancer cells via inhibition of mTOR and induction of ER stress and DNA damage [38]; therefore, sensitivity to radiation depends on many complex factors and variables. Interestingly, immunotherapy with high-dose interleukin IL-2 has been reported to give rise to systemic autophagic syndrome, but is able to produce long-term remission in certain groups of cancer patients [39]. In mouse models, inhibition of autophagy combined with high-dose IL-2 is able to enhance long-term tumor regression and alleviate toxicity [40].

### 2.2. Autophagy and Cancer Cell Death

Autophagy-dependent cell death (also called type II programed cell death) has been known to be caspase-dependent. Nevertheless, the crosstalk between autophagy and apoptosis is more complex in cancer cells. Some studies have elucidated that the cleavage of autophagic proteins emerges when cells switch autophagy toward apoptosis. Thus far, it has been demonstrated that the autophagic protein, particularly Atg5, is cleaved by calpain to become a truncated product with a relative molecular weight of 24 kDa, resulting in cytochrome c release-triggered caspase activation [77]. Another study completed by Norman et al. showed that Atg proteins are not only cleaved by calpain but also by cell death proteases in HeLa cells [78]. Their study validated that both Atg3 and Beclin1 are cleaved by caspase-3 and caspase-6 while Atg4, Atg7, and Atg9 can be cleaved by caspase-3. Consistent with their report, our previous study also illustrated that arginine deprivation can induce autophagy in melanoma cells. However, the arginine deprivation-induced autophagic process is aborted upon cleavage of Atg5 and Beclin1 by caspase when combined with tumor necrosis factor (TNF)-related apoptosis-inducing ligand (TRAIL) [79].

Previously, it has been mentioned that ER stress induced by BRAF inhibitor triggers autophagy for melanoma cell survival (Table 1). However, treatment with one of cannabinoids, ∆^9^-tetrahydrocannabinol (THC), is capable of inducing autophagy-dependent cell death via ER stress and inhibition of AKT/mTOR in glioma cells [48]. Even though the detailed mechanism whereby ER stress triggers autophagy-mediated cell death is not elaborated, autophagy-induced cell death subsequent to THC treatment is through caspases activation. Different from THC, the inhibitor targeting inhibitor of apoptosis proteins (IAPs) combined with chemotherapies including cisplatin and vincristine lead to concomitant induction of autophagy and apoptosis in medulloblastoma (MB) cells [72]. Treatment with autophagy inhibitor CQ abolished combination treatment-induced cell death, which further validated that combination treatments bring on autophagy–mediated cell death. Similar to this approach, treatment with bisphosphonates causing blockade of mevalonate pathway also initiate autophagy and apoptosis as evidenced by increased LC3-II and caspase-3 expressions in prostate cancer and salivary adenoid cystic carcinoma (SACC) cell lines [51,52]. Addition of autophagy antagonist 3-MA shows a decrease in bisphosphonate-induced apoptosis, and hence proves that bisphosphonate treatment in cancer cells is able to trigger type II programmed cell death. Although it has been confirmed that these agents induce autophagy-mediated cell death by autophagy inhibitors, how autophagy initiates cell death is still poorly understood.

## 3. The Interplay between Autophagy and Tumor Microenvironment

It has been known that the hostile tumor microenvironment influences autophagy in tumor cells, tumor infiltrating cells and vice versa. These microenvironmental factors include hypoxia, inflammation, and cytokines in tumor milieu [80]. In response to stresses in tumor microenvironment, autophagic process is activated to conserve/supply energy by digesting the intracellular components and prevents accumulation of toxic components in the cells. Nevertheless, the molecular basis whereby these factors provoked from tumor milieu influence autophagy still needs to be elucidated. These mechanisms are described below.

### 3.1. Hypoxia Induces Autophagy in Tumor Microenvironment

Several studies indicate that 50% of tumors thrive under hypoxia [81,82]. Hypoxic condition in tumor may have different effects on autophagy pathways upon duration and severity of oxygen depletion. Under moderate and chronic hypoxia, hypoxia-induced factor-1 alpha (HIF-1α) and PKCδ-JNK govern activation of autophagy [76,83]. JNK, as mentioned above, releases Beclin1 from BCL2 suppression/binding, and hence facilitates autophagosome formation (Table 1). On the other hand, hypoxia-induced autophagy is more likely through unfolded protein response (UPR) and ER stress pathway mediated by PERK–eIF2α–ATF4 [84]. Except for HIF-1α modulation in hypoxia-induced autophagy, hypoxia accompanied by glucose and amino acid deprivation results in HIF-independent autophagy triggered by AMPK activation and mTOR inhibition [83]. Interestingly, a recent study discovered that DJ-1 (also known as PARK7) is vital for transcription of HIF-responsive gene and AMPK activity. Deletion or knockdown of DJ-1 in U2OS cell line and MEFs led to low expressions of HIF-1α, AMPK, and LC-3-II during hypoxia [85]. Taken together, hypoxia triggers DJ-1, which regulates HIF-dependent and HIF-independent autophagy.

Since hypoxia results in REDD1- or BNIP3-mediated autophagy, as mentioned previously (Figure 1), studies have been carried out to examine whether there is a correlation between HIF-1α and BNIP3 or REDD1. Interestingly, the evidence indicated that HIF-1α can upregulate transcription of BNIP3. Elevated BNIP3 then interferes Beclin1 and BCL2 forming complex as well as suppresses Rheb-mTOR activation [7,86]. REDD1, the other protein induced by hypoxia, is able to dissociate the 14-3-3 proteins from the complex with TSC2, and consequentially reduces mTOR activity [87]. Furthermore, a stress sensor, ataxia telangiectasia mutated (ATM), has been demonstrated to participate in regulation of REDD1-modualted mTOR signal. Under hypoxic condition, ATM (−/−) MEFs displayed decreased expressions of HIF-1α and REDD1 [88]. Altogether, it is suggested that hypoxia-induced ATM activation results in upregulated HIF-1α–BNIP3 and REDD1 to attenuate mTOR activity and elicit autophagy.

### 3.2. The Effect of Inflammation on Autophagy in Tumor Milieu

The growing epidemiological evidence suggests a possible relationship between dysplasia and chronic or intermittent inflammation [80]. Several chronic inflammatory diseases including inflammatory bowel disease (IBD), pancreatitis, and hepatitis are known to promote malignant changes which lead to colon cancer, pancreatic cancer and hepatoma, respectively. When inflammation occurs, tumor cells, macrophages, and other tumor infiltrating immune cells possess high reactive oxygen species (ROS), and secrete chemokines and cytokines such as IL-6, tumor necrosis factor (TNF)-α, IL-1β, IL-10, and transforming growth factor (TGF)-β to tumor microenvironment [80]. IL-1β is also known to activate ROS production to promote NOD-like receptor family, pyrin domain containing 3 (NLRP3) inflammasome complex formation, which further amplifies the inflammatory response through secretion of more pro-inflammatory cytokines (e.g., IL-1β and IL-18). Using co-culture system of fibroblasts and tumor cells, a dramatic increase in inflammatory mediators including macrophage inflammatory protein 1-alpha (MIP-1α, also known as CCL3), granulocyte-macrophage colony-stimulating factor (GM-CSF), IL-6, IL-8, IL-10, interferon (IFN)-γ, and RANTES (also known as CCL5) as well as activation of NF-κB and HIF-1α signals can be elicited. All of these mediators and signals generate autophagic environment that includes autophagy in adjacent fibroblasts [89]. Among these inflammatory mediators, IL-6 and TNF-α have been reported to upregulate NF-κB activation of Kupffer cells in liver and in turn boost hepatocarcinogenesis [90]. Taken together, inflammation positively regulates autophagy in tumor microenvironment and tumor adjacent cells, promoting tumor initiation and progression.

### 3.3. The Impact of Autophagy on Tumor Promoting Inflammation

Some studies, as mentioned, have demonstrated that inflammation is one of the processes for tumorigenesis which reduces activity of autophagy; however, other studies indicated that autophagy, particularly mitochondria-selective autophagy (so-called mitophagy), suppresses inflammation through inhibition of NLRP3 inflammasome formation. This controversy stems from the fact that autophagic process, like other biochemical process, has its negative feedback loop to prevent constant hyper-inflammation-induced severe tissue damage and cell death [91]. Consistent with this notion, Atg16L1, an autophagic protein, has been proven to govern intestinal homeostasis and modulate susceptibility to Crohn’s disease, a chronic inflammation occurring in intestine. In mouse colitis models induced by dextran sodium sulfate, Atg16L1-deficient macrophages secreted a lot of pro-inflammatory cytokines including IL-1β and IL-18 via TRIF-activated caspase-1 after stimulated with lipopolysaccharide [92]. Similarly, conditional Atg7 knockout mice also exhibited impaired autophagy in alveolar macrophages, which resulted in activation of IL-1β-enhanced inflammasome formation and sequentially brought on pulmonary inflammation [93]. In oncogenic mutation-driven tumorigenesis, activation of PI3K-AKT-mTOR impairs autophagy and thereby enhances necrosis leading to inflammation which can accelerate tumor proliferation. Tumor necrosis gives rise to an increase in expression of inflammatory mediators including high mobility group box 1 (HMGB1), damage-associated molecular patterns (DAMPs), uric acid, and ATP/UTP. These cytokines and inflammatory mediators account for the recruitment of monocyte-macrophage lineage cells and other antigen presenting cells (APCs) which may favor T cell activation. Overall, these inflammatory mediators are important in tumor development and progression through maintaining malignant features such as cell proliferation, angiogenesis, and metastasis and hence are associated with poor prognosis [94].

In rare cell types, such as epithelial cells transformed with oncogenic RAS and breast cancer stem-like cells, depletion of autophagy associated gene, such as Atg7, Atg12, or FIP200 causes decreased pro-inflammatory cytokine IL-6 secretion and impaired Signal transducer and activator of transcription 3 (STAT3) or TGFβ/Smad pathways, resulting in abrogation of cell invasion and tumor initiation [95,96].

## 4. The Influence of Autophagy on Immune Surveillance

The mechanisms whereby autophagy manipulates anticancer immunity are diverse and remain controversial. According to the review article by Chen et al., initiation of anticancer immunity must go through several steps: (1) release of tumor neoantigen; (2) presentation of tumor neoantigen; (3) priming T cells; (4) recruitment of T cells to tumor; (5) recognition of tumor cells by T cells; (6) T cell-mediated cytotoxicity [97]. To trigger T cell activation, cancer cells release neoantigens caused by oncogenic mutations. These neoantigens are presented by cancer cells per se through major histocompatibility complex (MHC) I, or taken up by dendritic cells (DCs) to process for MHC II antigen presentation. DCs carrying antigens then circulate to lymph nodes to prime T cells. Antigens presented on the surface of cancer cells and DCs are recognized by T cell receptor (TCR) on cytotoxic CD8+ T cells and CD4+ T cells, respectively. After activated, CD8+ T cells infiltrate into tumors, and eliminate cancer cells expressing neoantigens by releasing INF-γ, perforin, and granzyme B. These processes need to be accompanied by specific signals (e.g., inflammatory cytokines including TNF-α, IFN-γ, and IL-1; other cytokines including IL-2 and IL-12), which overcome peripheral tolerance provided from immune suppressive cells, such as regulatory T (T_reg_) cells, myeloid-derived suppressor cells (MDSCs), tumor associated macrophages (TAMs) in tumor milieu.

**Figure 2:**
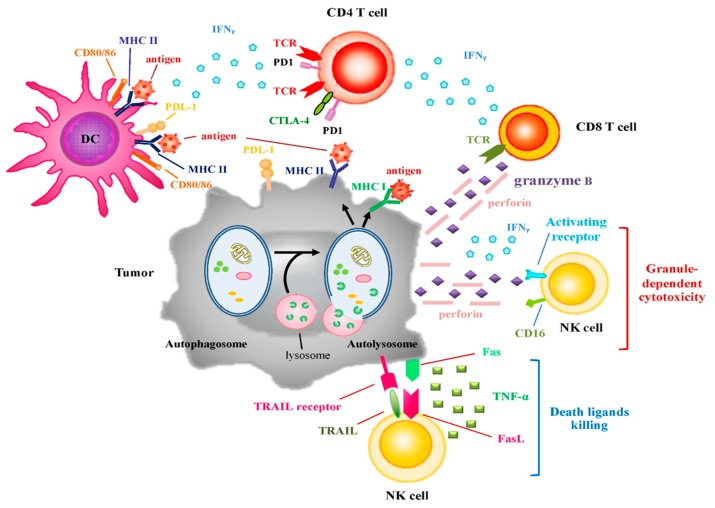
The role of autophagosome in tumor antigen presentation and anticancer immunity. Tumor cells expressing neoantigens that are loaded on major histocompatibility complex (MHC) I can be recognized by T cell receptors (TCR) on the surface of CD8+ T cells. CD8+ T cells secret granules containing granzyme B and perforin from cytosol to eliminate tumor cells. Many solid tumors do not possess MHC II; therefore, their neoantigens are usually absorbed by antigen presenting cells (APCs) like dendritic cells (DCs). These APCs process tumor antigens and present them on MHC II which can be recognized by CD4+ T cells to potentiate immunity via interferon (IFN)-γ releasing. Currently, it has been reported that autophagy contributes to antigen presentation by MHC I as well as MHC II following stimulation with IFN-γ. Similar to cytotoxic T cells, nature killer (NK) cells eradicate tumor cells by releasing granzyme B and perforin or through cell death ligands/receptors such as TNF-related apoptosis-inducing factor (TRAIL)/TRAIL receptor and Fas/Fas ligand (FasL).

In addition to anticancer activity of T cells, natural killer (NK) cell cytotoxicity also contributes to immune surveillance. More recently, NK cell cytotoxicity is known to be carried out by two mechanisms: granule-dependent cytotoxicity and death ligand killing [98]. The former mechanism is initiated by Fc receptor (CD16, essential for antibody-dependent cell-mediated cytotoxicity (ADCC) and activating receptors including NKp30, NKp44, NKp46, and NKG2D [99]. NKG2D can recognize its ligand NKG2DL, which has two families including MHC I chain-related molecules A and B (MICA/MICB) and UL16-binding proteins (ULBP). Once NK cells are stimulated, granules containing perforin and granzyme B in cytosol are polarized toward cell membrane. The latter mechanism is triggered by death receptors/ligands, such as Fas/Fas ligand (FasL) and TRAIL/TRAIL receptor. NK cells secrete TNF-α to enhance cytotoxicity after activated by these death ligands/ receptors (Figure 2) [100].

Based on the anticancer immune system described above, the role of autophagy in immunity focuses on antigen presentation in cancer cells or APCs, on cytotoxicity of T and NK cells, and on lysosomal exocytosis in the course of immunogenic cell death (ICD).

### 4.1. Autophagy and Immunogenicity

Autophagosmes play a crucial role in endogenous and exogenous antigens fusing with MHC I and MHC II for antigen presentation and recognition by TCRs on the surface of T cells (Figure 2). For instance, high levels of autophagic proteins seen in thymic epithelial cells enabled self-antigens to be loaded to MHC II and thereby facilitated CD4+ T cell selection [20]. Moreover, tumor cells utilize autophagosome formation to fuse endogenous and exogenous antigen process with MHC I and II for antigen presentation to T cells.

In regard to the paradigm of antigen processing for presentation of MHC II, it consists of α and β subunits assembled in the ER and coupled with invariant chain. Meanwhile, intracellular (nuclear and cytoplasmic) antigens are sequestered in double membrane autophagosomes or lysosomes that contain proteases to digest and denature antigens and in turn yield peptides. Peptides reach MHC II to form peptide-MHC II complexes in autolysosomes and are subsequently trafficked to the surface of APCs. For exogenous antigen, LC3-associated phagocytosis (LAP), a conjunction of LC3 and phagosomal membrane surrounding toll-like receptor-2 (TLR2), Fc receptor, and TIM-4, delivers them to endosomal MHC loading compartment [101,102]. Treatment with inflammatory cytokine IFN-γ can enhance MHC II expression through fusion with autophagosomes in APCs like DCs, epithelial cells, fibroblasts, and rare tumor cells [103].

Different from antigen processing for MHC II presentation, the role of autophagosome in antigen processing for MHC I presentation is not well studied. Briefly, intracellular antigens or self-antigens are denatured into peptides by proteasomes in cytosol [104], and then these peptides are translocated into ER lumen through transporter associated with antigen processing (TAP), which is a heterodimeric transporter binding to peptides. This process requires hydrolysis of ATP. In ER, peptides are loaded onto MHC I loading complex. This process is facilitated by several proteins including tapasin, ERp60, and calreticulin. Peptide-MHC I complexes are eventually delivered to the surface of cell membrane where the peptides are presented to TCR via vesicle transport [105]. For MHC I cross-presentation of exogenous antigens, similar to MHC II, APC cells engulf and sequester exogenous antigens in LC3-decorated phagolysosomes in cytosol [106]. This mechanism is also supported by a study showing that HEK293 cells expressing ovalbumin (OVA) antigen treated with mTOR inhibitor rapamycin underwent autophagy and displayed elevation of OVA cross-presentation of MHC I [107]. In contrast, treatment with autophagy antagonist 3-MA drastically can suppress OVA cross-presentation. Whether endogenous antigens processing for MHC I presentation is autophagy-dependent remains uncertain and requires further investigation. Recent study discovered that autophagy was capable of facilitating MHC I expression stimulated by IFN-γ in B16 mouse melanoma cells [108]. Another study also uncovered that silencing Atg5 using siRNAs failed to assist Herpes simplex virus-1 (HSV-1) antigen processing for MHC I presentation and CD8+ T cells activation [109]. Collectively, autophagy has been suggested to contribute to antigen processing for MHC I as well as MHC II presentation.

In the process of immunogenic cell death (ICD), dying cells release HMGB1, DAMP, and ATP, which exert their ability to recruit DCs to take up antigens from dying cells and then present them to T cells. Intriguingly, ATP storage in lysosome and the secretion process are controlled by autophagy. When the cells are in resting condition, autophagy is responsible for the maintenance of lysosomal ATP in the cells. In ICD, ATP release is modulated by lysosome and lysosome exocytosis-related proteins, such as lysosomal-associated membrane protein 1 (LAMP1) and pannexin1 (PANX1). In this regard, Wang et al. confirmed that depletion of Atg5 or Atg7 did not reduce ATP levels and disturb the function of PANX1 in the resting condition; however, in the course of ICD, the portions of LAMP-1 and lysosomal ATP in Atg5 or Atg7-depleted cells were less than that in autophagy-competent cells [110]. Therefore, autophagy is required for maintenance of lysosomal ATP and ATP release in ICD, and consequently promotes T cell activation.

### 4.2. The Roles of Autophagy in Immune Cell Cytotoxicity

Autophagy has been regarded as a survival mechanism or suppression of proliferation in many cell types. In the development of T cells, ablation of autophagic proteins such as class III PI3K, Beclin-1, Vps34, Atg5, and Atg7 resulted in abrogation of T cell survival due to failure of ER homeostasis, mitochondrial clearance, and increased levels of ROS [111,112,113,114]. Previous study utilized Atg5 (−/−) chimeric mice, that is, mice received irradiation and their immune system was reconstituted by transferring fetal liver cells from Atg5 (−/−) donors; the result revealed that Atg5 (−/−) T cells fully matured but failed to undergo efficient proliferation following TCR stimulation. Peripheral Atg5 (−/−) CD8+ T cells exhibited a drastic increase in cell death [115]. Thus, autophagy is suggested to govern T cells proliferation and survival. Paradoxically, T helper 2 (Th2) cells were prone to undergo autophagy more than Th1 cells. Using autophagy inhibitor 3-MA or RNAi against Beclin-1 or Atg7 to suppress autophagy prevented growth factor-withdrawal cell death but did not affect proliferation in Th2 cells. Hence, autophagy-mediated cell death accounts for Th2 cell homeostasis. Overall, the role of autophagy in T cell development may be different from that in T cell differentiation.

While autophagy contributes to T cell development, hypoxia-induced autophagy results in resistance to T cell cytotoxicity due to activation of STAT3. Noman’s group further discovered that inhibition of autophagy by silencing Beclin1 or Atg5 correlated with decreased hypoxia-induced phosphorylated STAT3, which can render lung cancer cells sensitive to T cell cytotoxicity [116]. Aberrant activation of STAT3 can reduce immune response by suppressing IFN-γ, TNF and CXCL10 expression, participating in DC maturation and T cell activation. Nevertheless, the crosstalk between STAT3 activation and autophagy induced by hypoxia still needs clarification. On the other hand, another study also showed that granzyme B released from NK cells can be degraded by autophagosomes in hypoxic breast cancer cells. Depletion of Beclin1 or Atg5 enabled breast cancer cells to regain susceptibility to NK cell cytotoxicity [117]. Additionally, hypoxia-induced autophagy enhances degradation of gap junction protein connexin 43 (Cx43). Cx43 has been proven to enhance interaction between target and effector cells. Inhibition of autophagy in melanoma cells led to an increase in Cx43 protein levels and thereby enhanced cytotoxic activity of NK cells [118]. Altogether, hypoxia-induced autophagy assists tumor cells to evade cytotoxic immune response.

### 4.3. Autophagy and Immune Checkpoints

Immune checkpoints, such as program death ligand1/2 (PD-L1/L2), galectin-9 (GAL-9), and CD80/86 (also known as B7-1/2), are commonly seen in tumor cells, tumor associated macrophages, DCs, and MDSCs. These proteins can separately interact with program death-1 (PD-1), TIM-3, and cytotoxic T lymphocyte associated protein 4 (CTLA-4) located on the surface of T cells (Figure 2). Activation of these checkpoints dampens T cell activation and causes T cell exhaustion which helps tumor cells evade from immune surveillance. To enhance anti-cancer immunity, the inhibitors targeting these immune checkpoints, such as anti-PD-1 (nivolumab and pembrolizumab), anti-PD-L1 (atezolizumab, avelumab, and durvalumab), and anti-CTLA4 (Ipilimumab) antibodies, have been developed and are FDA-approved to treat melanoma, non-small cell lung cancer (NSCLC), bladder cancer, head and neck cancer, kidney cancer, and other cancer types [119]. TIM-3, which has been found on 30% of CD8 tumor infiltrating lymphocytes (TILs) in NSCLC, has emerged as an immune checkpoint related to resistance to anti-PD-1 inhibitors [120]. Thus, anti-TIM-3 antibody is developed to improve anticancer immunity of anti-PD-1 blocking antibody.

Currently, there are few studies implicating a direct interplay between autophagy and immune checkpoints expression in tumor cells. It is of note that among these immune checkpoints, PD-L1 expression in NSCLC is upregulated by oncogenic activation of the AKT-mTOR pathway and mTOR-mediated IFN-γ expression [121]. Despite the fact that inhibition of mTOR can result in initiation of autophagy and downregulation of PD-L1 expression, it remains unclear whether autophagy regulates PD-L1 expression. Interestingly, in murine B16 melanoma and ID8agg ovarian cancer cell lines which express PD-L1, blockade of PD-L1 using RNAi can retard proliferation and is attributed to augmentation of genes related to autophagy. Indeed, robust autophagy induced by PD-L1 attenuation sensitizes cancer cells to autophagy antagonists [122]. Therefore, PD-L1 expression can somehow contribute to downregulation of autophagy.

In regard to the engagement of PD-1 in T cells, it suppresses stimulation of TCR/CD3 and CD28-enhanced glucose uptake and glycolysis by decreasing expression of glucose transporter Glut1. Additionally, the energy sensor, AMPK–ULK1 axis, was dramatically activated in T cells after receiving PD-1 signal, but unchanged in the absence of PD-1 [123]. Consistent with these signaling events, T cells when receiving PD-1 signal during stimulation of TCR/CD3 and CD28 also highly expressed LC3-II, a well-characterized marker of autophagosome formation. Overall, PD-1 ligation reprograms T cell metabolism by suppressing glycolytic pathway and promoting glucose starvation-induced AMPK–ULK1 activation; thereby triggering autophagy in T cells.

## 5. Autophagy Paradox: Activating Autophagy by Therapeutic Agents is Pros or Cons for Anti-Cancer Immunity?

Autophagy contributes to intrinsic signals (e.g., cell survival, cell death, cell proliferation, immunogenicity) and extrinsic signals (e.g., cross-presentation of antigens, T cell activation, and cytotoxicity of immune cells) in tumor cells. Most of anti-cancer therapies can trigger autophagic flux and display different anti-cancer effects in various cancer types. However, in clinic, whether anti-cancer agent-elicited autophagy can alter immune system and thus inhibition of autophagy influences anti-tumor effect of immunotherapies is still under debate. In this respect, several factors acting in concert with autophagy in anticancer immunity are also needed to be considered. The first factor is whether these therapies can induce ICD (immunogenic cell death) to release tumor antigen, and in turn activate cytotoxic T cells through cross-presentation of DCs. The second factor is whether tumor antigens can be presented through increased MHC I and MHC II expressions following treatment with anticancer agents. The third factor is whether these treatments can potentiate T cell and NK cytotoxicity. The fourth factor is whether these therapeutic agents can reduce expression of immune checkpoints, particularly PD-L1. The fifth factor is whether these therapies can suppress recruitment of immunosuppressive cells to tumor milieu by reducing the levels of immunosuppressive factors. These factors are the indexes for assessment of anti-cancer immunity described below.

### 5.1. Anticancer Treatments-Induced Autophagy and Immune System

As mentioned, numerous in vitro and in vivo studies supported that autophagy contributes to cell survival and thereby renders tumor cells resistant to certain anti-cancer agents. Moreover, some anti-cancer agents also manipulate the immune system. According to the review articles written by Chacon et al. [124] and Bezu et al. [125], some commonly used antitumor agents in the clinic, such as bortezomib, cyclophosphamide, doxorubicin, and SAHA, employ ICD to enhance DC maturation and anticancer activity of T cells (Table 2). The process of ICD, as described previously, requires autophagy to release mediators like ATP, resulting in DC recruitment. Certainly, treatment with these agents, except bortezomib, supports that induction of ICD may be enhanced by autophagy or mediated through autophagy [124]. In addition, consistent with previous reports illustrating that autophagy is implicated in antigen processing for MHC presentation, concomitant induction of autophagy and MHC expression can be seen in cancer cells when treated with the conventional chemotherapies (e.g., cisplatin, taxol, and gemcitabine), targeted therapeutic agents (e.g., vemurafenib and trametinib), radiotherapy, and treatment with interferons (type I/II). Taken together, these evidences suggest that therapies-induced autophagy may contribute to anti-cancer immunity. On the other hand, treatments such as cyclophosphamide, 5-FU, and arsenic trioxide, and interferons can enhance NK activation due to elevation of activating receptors and their ligands. Rapamycin, different from other therapeutic agents, has been proven to induce autophagy via mTOR inhibition in cancer cells; nevertheless, it also has been considered as an immunosuppressive drug because of its ability to impair DC maturation and T cell proliferation. It is reasonable due to the fact that both DC and T cells rely on mTOR instead of autophagic protein to accomplish their maturation and differentiation [126]. Recent study discovered that mTOR inhibitor rapamycin can attenuate influx of tumor infiltrating T_reg_ cells [127], yet reduction of T_reg_ influx may not be sufficient enough to overcome impaired DC and T cell activation and differentiation.

With respect to immune checkpoint PD-L1 expression, among these treatments, only treatment with vemurafenib or in combination of trametinib or treatment with rapamycin reduces the expression of PD-L1 (Table 2). These evidences are in accordance with other studies illustrating that PD-L1 expression is governed by oncogenic activation of AKT-mTOR in glioma cells with PTEN deletion, and in lung cancer cells with EGFR or KRAS mutation [121,148,149]. Interestingly, BRAF (V600E) mutation in melanoma cells does not evidently affect PD-L1 expression; however, PD-L1 expression is attenuated by treatment with BRAF inhibitor, but enhanced when melanoma cells become resistant to BRAF inhibitor [130]. We have also previously shown that resistance to BRAF inhibitor contributes to decreased levels of autophagic proteins and abrogation of autophagy [34]. Therefore, it is highly suggestive that PD-L1 expression is conversely related to induction of autophagy. Nevertheless, in some circumstance, PD-L1 expression may result from autophagy. As shown in Table 2, treatment with cisplatin, taxol, gemcitabine, 5-FU, arsenic trioxide, or radiation results in concomitant induction of autophagy and PD-L1 expression. One possibility is that tumor cells acquire autophagy when they undergo epithelial to mesenchymal transition (EMT) during metastasis in order to survive and to become resistant to these anticancer treatments. They also need to express PD-L1 to escape from T cell cytotoxicity during their metastatic process [150,151,152]. Furthermore, recent study found that PD-L1 transcription can be upregulated by zinc finger E-box-binding homeobox 1 (ZEB1), which mainly contributes to EMT [153]. This is supported by the latest evidence revealing that carcinoma cells with mesenchymal feature possess lower MHC I and higher PD-L1 expressions while epithelial carcinoma cells possess opposite expressions [154]. Collectively, it is suggested that autophagic process may be found with elevated PD-L1 expression and diminished MHC I which protect mesenchymal carcinoma cells from immune attack.

Since PD-L1-mediated T cell exhaustion emerges following anticancer treatments, some studies proposed combination of PD-1/PD-L1 blockade with conventional therapies to unleash cytotoxic T cells as well as to inhibit tumor cell proliferation. For instance, the clinical trials of anti-PD-1 antibody (pembrolizumab or nivolumab) combined with chemotherapy (cisplatin, carboplatin, gemcitabine, bevacizumab, or paclitaxel) for NSCLC are ongoing [155]. In fact, one of the trials, combining pembrolizumab with pemetrexed and platinum, has produced a significant survival benefit compared to chemotherapy, and has received FDA approval as front line therapy for metastatic lung adenocarcinoma [156]. In mouse pancreatic ductal adenocarcinoma models, radiotherapy combined with anit-PD-L1 antibody significantly prevented liver metastasis and improved anticancer response evidenced by upregulated T cell activation markers (CD69, CD44, and FasL) as well as and increased CD8:T_reg_ ratio [157].

### 5.2. Are Autophagy Antagonists Suitable to Combine with Immunotherapies?

Autophagy has a dual-faceted function regulating immune response and creating a set of challenges to improve anticancer immunity. Some studies described above have suggested that conventional anticancer treatment-induced autophagy could benefit anticancer immunity (Table 2), whereas numerous studies have demonstrated that autophagy confers resistance to conventional anticancer therapies and circumvents cell death. Hence, whether autophagy inhibition is pro or con when combined with immunotherapies to treat cancer patient remains controversial. This may also be governed by cancer types and immune systems in tumor milieu. Given all the available evidence, which showed that autophagy leads to elevated STAT3 activation favoring tumor cell survival and evading immune system, increased ability to degrade cytotoxic granules released from NK cells, and upregulation of PD-1–PD-L1 ligation-suppressed T cell proliferation, autophagy antagonists are pro for anticancer immunity. The noteworthy example supporting this notion is combination of CQ and high dose IL-2 to mitigate systemic autophagic syndrome and promote cancer cell apoptosis in animal models, yet the clinical trial of this combination treatment is still ongoing [40]. Nevertheless, the result of the phase II clinical trial showed that autophagy inhibitor CQ does not exhibit significant improvement in overall survival while combined with radiation and temozolomide to treat glioma patients [158]. Other clinical trials of autophagy antagonists combined with conventional treatments are still ongoing. Inhibition of autophagy theoretically is con for DC and T cell maturation and differentiation due to inactivation of ICD. Surprisingly, Starobinets et al. disclosed an important finding that doxorubicin combined with inhibition of autophagy (treatment with CQ or genetic knockdown of Atg7 or Atg12) elicited the intact T cell function and activation as well as those in doxorubicin-treated autophagy-competent tumors [159]. Altogether, these results imply that autophagy antagonists augmented anti-cancer effect of chemotherapy by inhibiting cell survival and maintaining immunity instead of attenuating ICD and immunogenicity.

## 6. Concluding Remarks

Autophagy is a catabolic process that tightly correlates with cellular program by engulfing and sequestering intracellular components into lysosomes to maintain cell homeostasis. In the past decade, many studies investigated how autophagy influences cancer cell survival or death and resistance to therapeutic agents as summarized above. Other studies also examined how to effectively combine autophagy antagonists with current treatments to overcome resistance. Until recently, immune checkpoints have been discovered to hamper anticancer immune response in certain subset of cancers. Blockade of immune checkpoints has exhibited a great promising improvement of anticancer effect of current therapies. While seeking for optimal combination treatments to achieve enhancement of immune response and abrogation of autophagy-mediated drug resistance, it is intriguing how autophagy influences anticancer immunity. In this regard, autophagy functions not only as stress response to prevent cell death, but also as antigen processing for presentation to cytotoxic T cells and immune cell differentiation and development. Based on this reason, activating autophagy not only affects cancer cells but also manipulates features of immune cells and stromal cells in tumor milieu. Numerous evidences supported that anticancer therapies-initiated autophagy seems to increase tumor immunogenicity that can boost cytotoxicity of T and NK cells, yet several evidences have illustrated that inhibition of autophagy does not subvert anticancer immunity. Therefore, the effect of targeting autophagy on cancer cells and immune system may be beyond immunogenicity and activation of immune cells. Some complex factors involved in immunity and tumor microenvironment including hypoxia, tumor infiltrating immunosuppressive cells, tumor associated fibroblasts, EMT, and other unidentified factors need to be considered for the future direction in this area.

## Figures and Tables

**Table 1:** Current and prospective treatments result in autophagy-mediated cancer cell death or survival.

Treatment	Action	Autophagy Role	Cancer Type	Reference
Radiation 5-FU	DNA damage, ER stress, mTOR inhibition Thymidylate synthase inhibitor	Survival Death Survival	Glioma (CSC), Esophageal Glioma, Cervical Colon, Esophageal	[41,42] [43,44] [27,45]
Temozolomide	DNA damage	Survival	Glioma	[26]
Cisplatin	DNA damage	Survival	Esophageal, Cervical, Melanoma, Ovarian, Lung	[27,28,29,30,31]
SAHA	HDAC inhibitor	Survival	CML	[46]
Gemcitabine	DNA synthesis inhibitor	Survival	Lung	[32]
		Death	Pancreatic	[47]
Cannabinoids	ER stress	Death	Glioma, HCC, Melanoma	[48,49,50]
Bisphosphonates	Farnesyl pyrophosphate synthase inhibitor, mevalonate	Death	Prostate, SACC	[51,52]
NVP-BEZ235	PI3K/AKT/mTOR inhibitor	Survival	HCC, Mesothelioma, Lung	[53,54,55]
		Death	Prostate	[56]
RAD001 (Rapamycin derivative)	mTOR inhibitor	Survival	Bladder	[57]
		Death	Prostate, ALL	[58,59]
AZD8055	mTORC1/mTORC2 complex inhibitor	Survival	Colon	[60]
		Death	HCC	[61]
Erlotinib, Gefitinib	EGFR mutation inhibitor	Survival	Lung	[62,63]
Cetuximab	EGFR inhibitor	Survival	Lung, Epidermoid carcinoma	[64]
Sorafenib	Tyrosine kinase inhibitor	Survival	Colon, HCC	[65,66]
Imatinib	Tyrosine kinase inhibitor	Survival	Glioma, CML	[67,68]
Vemurafenib/Dabrafenib	BRAF (V600E) inhibitor, ER stress	Survival	Melanoma, Glioma	[69,70]
Trastuzumab	HER2 inhibitor	Survival	Breast	[71]
LCL161/LBW242 + Vincristine or Cisplatin	IAPs inhibitor + Tubulin inhibitor or DNA damage	Death	MB	[72]
2-DG	Glycolysis inhibitor, ER stress	Survival	Prostate, Pancreatic, Melanoma, Breast	[73,74]
ADI-PEG20/Arginine deiminase, Arginase	Arginine depletion	Survival	Melanoma, Prostate, Sarcoma,	[18,75,76]
IL-2	Immunotherapy	Survival	Colon, Pancreatic	[40]

5-FU: 5-fluorouracil; ER: endoplasmic reticulum; CSC: cancer stem cell; mTOR: the mechanistic target of rapamycin; HDAC: histone deacetylases; CML: chronic myeloid leukaemia; SACC: salivary adenoid cystic carcinoma; ALL: acute lymphoblastic leukemia; EGFR: epidermal growth factor receptor; HCC: hepatoceulluar carcinoma; IAPs: inhibitor of apoptosis proteins; MB: medulloblastoma; 2-DG: 2- deoxyglucose; ADI: arginine deiminase; IL-2: interleukin-2.

**Table 2:** The influence of autophagy induced by current anticancer agents on immunity.

Treatment	Induction of Autophagy	Pros to Immunity	Cons to Immunity	Reference
Rapamycin	mTOR inhibition	Reduced T_reg_ influx and PD-L1	Impaired DC maturation and T cell differentiation	[121]
Bortezomib	ER stress	Increased ICD, DC maturation, NK activation	Decreased MHC I	[128]
Cyclophosphamide	ER stress	Increased ICD, MHC, TRAIL CD8 T cells, NK, and DC activation T_reg_ depletion		[124]
Trametinib	ER stress	Increased MHC and T cell proliferation , decreased PD-L1 and immunosuppressive factors	Increased PD-L1 when becoming resistant	[70,129,130]
Vemurafenib	ER stress	Increased MHC and T cell proliferation, decreased PD-L1 and immunosuppressive factors	Increased PD-L1 when becoming resistant	[70,129,130]
Cisplatin	ATM	Increased MHC, HMGB1	Increased PD-L1	[124,131]
Taxol	UPR	Increased MHC, decreased MDSC activation	Increased PD-L1	[124,132,133]
Gemcitabine	NF-κB	Increased MHC, NK activation via MICA	Increased PD-L1	[124,134]
Doxorubicin	AMPK–ULK1	Increased ICD and antigen presentation of DC		[135,136]
5-FU	BNIP3	Increased NK and T cell cytotoxicity while combined with interferons	Increased PD-L1	[137,138]
SAHA	mTOR inhibition	Increased ICD and TRAIL, DC and T cell activation		[139,140,141]
Arsenic Trioxide	mTOR inhibition	Increased NK activation via MICA/B and ULBP	Increased PD-L1	[124,142]
Radiation	Arg4B	Increased MHC I, IFN-β mediated cross-presentation of DC, decreased T_reg_ activity		[143,144,145]
Interferons	Beclin1	Increased MHC, NK and T cell activation	Increased PD-L1	[146,147]

mTOR: the mechanistic target of rapamycin; ER: endoplasmic reticulum; ICD: immunogenic cell death; DC: dendritic cell; NK: natural killer; MHC: major histocompatibility complex; PD-L1: program death ligand 1; ATM: ataxia telangiectasia mutated; UPR: unfolded protein response; MDSC: myeloid-derived suppressor cell; MICA: MHC I chain-related molecules A; AMPK: 5′AMP-activated protein kinase; ULK1: Unc-51-like kinase 1; 5-FU: 5-fluorouracil; BNIP3: BCL2 interacting protein 3; TRAIL: TNF-related apoptosis-inducing factor; ULBP: UL16-binding proteins; IFN: interferon.

## Abbreviations

ADCC	Antibody-dependent cell-mediated cytotoxicity
ADI	Arginine deiminase
ALL	Acute lymphoblastic leukemia
AMPK	5′ AMP-activated protein kinase
ASS1	Argininosuccinate synthase 1
ATF4	Activating transcription factor 4
Atg	Autophagy-related protein
ATM	Ataxia telangiectasia mutated
ATP	Adenosine triphosphate
BNIP3	BCL2 interacting protein 3
CASTOR1	Arginine sensor for mTORC1
CCL	CC chemokine ligand
CTLA4	Cytotoxic T lymphocyte associated protein 4
CMA	Chaperone-mediated autophagy
CML	Chronic myeloid leukemia
CQ	Chloroquine
CSC	Cancer stem cells
Cx43	Connexin 43
DAMPs	Damage-associated molecular patterns
DC	Dendritic cell
eIF-2α	α subunit of eukaryotic translation initiation factor 2
EGFR	Epidermal growth factor receptor
EMT	Epithelial to mesenchymal transition
ER	Endoplasmic reticulum
GAL-9	Galectin-9
GAP	GTPase activating protein
GM-CSF	Granulocyte-macrophage colony-stimulating factor
HCC	Hepatoceulluar carcinoma
HCQ	Hydroxy-chloroquine
HDAC	Histone Deacetylase
HER2	Human epidermal growth factor receptor 2
HIF-1α	Hypoxia-induced factor-1 alpha
HMGB1	High mobility group box 1
Hsc70	Cytosolic heat shock protein of 70 Kd
IAP	Inhibitor of apoptosis protein
IBD	Inflammatory bowel disease
ICD	Immunogenic cell death
IL	Interleukin
IRE1	Inositol-requiring enzyme 1
IRS-1	Insulin receptor substrate-1
JNK	Jun N-terminal kinase
LAMP-2A	Lysosome-associated membrane protein-2A
LAP	LC3-associated phagocytosis
LKB1	Liver kinase B1
MAPK	Mitogen-activated protein kinase
MB	Medulloblastoma
MDSC	Myeloid-derived suppressor cell
MEFs	Mouse embryonic fibroblasts
MHC	Major histocompatibility complex
MICA/B	MHC I chain-related molecules A and B
MIP-1α	Macrophage inflammatory protein 1-alpha
mTOR	Mammalian target-of-rapamycin
mTORC	Mammalian target-of-rapamycin complex 1
NK	Nature killer
NLRP3	NOD-like receptor family, pyrin domain containing 3
NSCLC	Non-small cell lung cancer
OVA	Ovalbumin (OVA)
PANX1	Pannexin1
PARK7	Parkinsonism associated deglycase
PD-1	Program death-1
PD-L1/2	Program death ligand 1/2
PDK1	Phosphoinositide-dependent kinase-1
PERK	Protein kinase RNA-like endoplasmic reticulum kinase
PTEN	Phosphatase and tensin homologue
Rag	Ras-related GTPase
REDD1	Rregulated in development and DNA damage responses 1
Rheb	Ras homolog enriched in brain
RNAi	RNA interference
ROS	Reactive oxygen species
SACC	Salivary adenoid cystic carcinoma
SAHA	Suberoylanilide hydroxamic
SQSTM1	Sequestosome 1
STAT3	Signal transducer and activator of transcription 3
TAM	Tumor associated macrophage
TAP	Transporter associated with antigen processing
TCR	T cell receptor
TGF-β	Transforming growth factor-beta
TILs	Tumor infiltrating lymphocytes
TIM-4	T cell immunoglobulin- and mucin-domain-containing molecule-4
TSC1/2	Tuberous sclerosis complex 1/2
TRAIL	TNF-related apoptosis-inducing factor
T_reg_	Regulatory T cells
TRIF	Toll/IL-1 receptor domain-containing adaptor inducing IFN-beta
ULBP	UL16-binding proteins
ULK1	Unc-51-like kinase 1
UPR	Unfolded protein response
ZEB1	Zinc finger E-box-binding homeobox 1
3-MA	3-methyladenine
5-FU	5-fluorouracil
